# A pan-cancer analysis of collagen VI family on prognosis, tumor microenvironment, and its potential therapeutic effect

**DOI:** 10.1186/s12859-022-04951-0

**Published:** 2022-09-27

**Authors:** Xiang Li, Zeng Li, Shanzhi Gu, Xinhan Zhao

**Affiliations:** 1 Department of Oncology, The First Affiliated Hospital of Xi’an Jiaotong University, NO.277, West Yanta Road, Xi’an, 710061 Shaanxi China; 2 Department of Second Medical Oncology, The 3201 Affiliated Hospital of Xi’an Jiaotong University, Hanzhong, Shaanxi China; 3 Department of Forensic Medicine, Xi’an Jiaotong University, NO.76, West Yanta Road, Xi’an, 710061 Shaanxi China

**Keywords:** Collagen VI family, Pan-cancer, Immune subtypes, Tumor microenvironment

## Abstract

**Background:**

Collagen VI family (COL6A) is a major member of extracellular matrix protein. There is accumulating evidence that COL6A is involved in tumorigenesis and tumor progression. In this study, we performed a systematic analysis of COL6A in pan-cancer based on their molecular features and clinical significance.

**Methods:**

Based on updated public databases, we integrated several bioinformatics analysis methods to investigate the expression levels of COL6A as well as the relationship between their expression and patient survival, immune subtypes, tumor microenvironment, stemness scores, drug sensitivity, and DNA methylation.

**Results:**

The expression levels of COL6A members varied in different cancers, suggesting their expression was cancer-dependent. Among COL6A members, COL6A1/2/3 were predicted poor prognosis in specific cancers. Furthermore, COL6A1/2/3 expression levels revealed a clear correlation with immune subtypes, and COL6A1/2/3 were associated with tumor purity, that is, gene expression levels were generally higher in tumors with higher stromal scores and immune scores. COL6A1/2/3 had a significantly negative correlation with RNA stemness scores, and meanwhile they were also related to DNA stemness scores in different degrees. In addition, the expression of COL6A1/2/3 was significantly related to drug sensitivity of cancer cells. Finally, our study revealed that COL6A1/2/3 expression was mainly negatively correlated with gene methylation, and the methylation levels showed remarkable differences in various cancers.

**Conclusions:**

These findings highlight both the similarities and differences in the molecular characteristics of COL6A members in pan-cancer, and provide comprehensive insights for further investigation into the mechanism of COL6A.

**Supplementary Information:**

The online version contains supplementary material available at 10.1186/s12859-022-04951-0.

## Background

Cancer is the leading cause of mortality worldwide and frequently displays heterogeneity and similarity in many morphological, biochemical, and physiological features [1–3]. Tumor microenvironment (TME) contains various cell types, surrounding stroma, and extracellular matrix (ECM), and its heterogeneity significantly influences therapeutic effect and clinical outcomes [4]. ECM, the scaffold of TME, regulates the composition of TME and promotes the occurrence and development of tumors [5]. Collagen type VI family (COL6A) is a major ECM protein mainly found in the basement membrane region. The major collagen VI isoforms comprise three polypeptide chains, α1 (VI), α2 (VI), and α3 (VI), which are designated by COL6A1, COL6A2, and COL6A3, respectively. In 2008, further studies identified another three additional collagen VI subunits encoded by COL6A4, COL6A5, and COL6A6 [6, 7]. In humans, COL6A4 is a non-processed pseudogene because it has been disrupted by an evolutionary pericentric inversion, and COL6A4 on chromosome 3 is broken into two pieces COL6A4P1 and COL6A4P2 [6]. COL6A forms a discrete network of beaded microfilaments that interact with other ECM molecules and provide structural support for cells, thereby contributing to the properties of the local ECM microenvironment [8]. Furthermore, COL6A also plays an indispensable role in binding to a range of cell surface receptors, and promoting the adhesion, proliferation, migration, and inflammatory responses of various cancer cell types [9–11]. It is clear that the signaling role of COL6A is very important in tumors.

Recent studies have shown that COL6A can influence tumor progression by directly stimulating tumor cells. Collagen VI deficiency (col6 − / −) dramatically reduces primary mammary tumor growth in mice [12]. COL6A1 increases tumor cell proliferation in osteosarcoma [11]. Furthermore, COL6A affects tumor metastasis. For instance, COL6A1 promotes vascular invasion and distant metastasis in pancreatic carcinoma [13]. COL6A1 is highly expressed in non-small cell lung cancer tissue samples with bone metastases, and overexpressed COL6A1 in lung cancer cells increases the adhesion of these cells to osteoblasts [14]. COL6A1 and COL6A2 have been observed to be significantly associated with invasion and metastasis by inhibiting the activities of MMP-2 and MMP-9 in bladder cancer cells [15]. COL6A6 suppresses the metastasis of pituitary adenoma via blocking the PI3K-Akt pathway [16]. Moreover, COL6A is also involved in TME to regulate tumor vascular remodeling and to promote tumor inflammation by recruiting macrophages [17]. Although our understanding of the role of COL6A in tumorigenesis has deepened over the past few years, however, a systematic understanding of COL6A members and their roles in prognosis, TME or therapy is still lacking.

In order to develop an integrated picture of commonalities and differences across tumor lineages, The Cancer Genome Atlas (TCGA) has proposed the Cancer Genome Atlas Pan-cancer Analysis Project in 2012 [18]. Pan-cancer analysis not only evaluates molecular aberrations and their functional roles in different tumor types, but also reveals the way to extend treatments that are effective in one cancer to another with a similar genomic profile. Therefore, we expanded our research scope to a pan-cancer analysis of COL6A members in 33 TCGA cancers. Additionally, the associations between COL6A gene expression and overall survival, immune subtypes, stemness scores, TME, drug sensitivity, DNA methylation, and miRNA-regulated network were investigated. Subsequently, the results obtained from TCGA data were further verified in independent colorectal cancer samples. Based on public resources and bioinformatics analyses, the similarities and differences in molecular feature and clinical significance of COL6A members, especially COL6A1/2/3, were comprehensively analyzed in pan-cancer.

## Methods

### TCGA pan-cancer data downloading

TCGA pan-cancer data are publicly available and they were downloaded from the UCSC Xena database (http://xena.ucsc.edu/). Gene expression RNA-seq (HTSeq-FPKM), phenotype information, and survival data were derived from GDC TCGA sets. Besides, immune subtypes and stemness scores based on DNA methylation (DNAss) and mRNA (RNAss) were collected from TCGA pan-cancer sets. We analyzed a total of 11,057 samples from 33 tumors in TCGA, including ACC, BLCA, BRCA, CHOL, CESC, COAD, DLBC, ESCA, GBM, HNSC, KICH, KIRC, KIRP, LAML, LGG, LIHC, LUAD, LUSC, MESO, OV, PAAD, PCPG, PRAD, READ, SARC, SKCM, STAD, TGCT, THCA, THYM, UCEC, UCS, and UVM. The detail of tumors and their corresponding normal samples and for the cancer type abbreviations please refer to Additional file 1: Table S1.

### Expression and coexpression analyses

For each tumor in TCGA, R-related packages were used to transform and integrate raw datasets of COL6A gene transcription Next, the normal group data were deleted and mRNA expression of COL6A members in 33 TCGA cancer types was visualized by box plots. Furthermore, “Wilcoxon test” was applied to analyze the differential gene expression of COL6A between the tumor group and the normal group using linear mixed effects models, and only 18 cancers with more than 5 associated adjacent normal tissue samples were included. Genes with a threshold of |log2 fold change (FC)|≥ 1 and *p*-value < 0.05 were identified as differentially expressed genes. Eventually, the correlation of COL6A members were performed by the R package “corrplot”, and Spearman’s correlation analysis was used as the statistical approach.

The Human Protein Atlas (HPA) database (https://www.proteinatlas.org/) was used to explore the mRNA expression profile distribution of COL6A in normal tissues and their protein expression patterns in tumor tissues [19]. The Cancer Cell Line Encyclopedia (CCLE) database (https://portals.broadinstitute.org/ccle) was used to investigate the mRNA expression profile of COL6A in cancer cell lines [20].

### Overall survival analysis

To explore whether the expression of COL6A was associated with patients’ overall survival in pan-cancer, the Cox proportional-hazards regression models were applied to examine hazard ratio (HR) of each COL6A member in 33 tumor types, and then the forest plot was delineated using the R packages “survival” and “forestplot”. COX *p*-value less than 0.05 was set as a threshold. In addition, patients were divided into high and low expression groups based on the median expression level of each COL6A member, and Kaplan–Meier survival curve was drawn using the R-package “survminer” and “survival” according to high and low risk values. The log-rank test was applied to analyze the differences in survival between the two groups. Statistical significance was defined as a *p*-value < 0.05.

### Correlation analysis of COL6A gene expression with immune subtypes, TME, and stemness scores

For 33 TCGA tumors, we accessed differential expression of COL6A members in the six immune subtypes, including C1 (wound healing), C2 (IFN-γ dominant), C3 (inflammatory), C4 (lymphocyte depleted), C5 (immunologically quiet), and C6 (TGF-β dominant) [21]. The R packages “limma”, “reshape2”, and “ggplot2” with the Kristal test were used to conduct analyses of immune subtypes. Then, we explored the infiltration levels of stromal cells and immune cells in 33 TCGA cancers, and R packages “estimate” and “limma” were applied to calculate stromal scores, immune scores, and estimate scores [22]. Finally, stemness scores including RNA stemness scores (RNAss) and DNA stemness scores (DNAss) were calculated by one-class logistic regression (OCLR) algorithm [23]. The correlation between the expression of COL6A members and scores was analyzed by the Spearman’s method. The *p*-value < 0.05 was considered statistically significant.

### Drug sensitivity analysis

The data including the mRNA expression of COL6A members and z scores for drug sensitivity were retrieved from the same sample of the NCI-60 cell line from nine different cancers. All data were downloaded from the CellMiner database (https://discover.nci.nih.gov/cellminer/) [24]. Then, we filtered drugs approved by FDA or used for validation in clinical trials. R packages “limma”, “ggplot2”, and “ggpubr” were used to process and visualize data. The association between gene expression and drug sensitivity was conducted by Pearson’s correlation test. The *p*-value < 0.05 indicated statistical significance.

### DNA methylation and miRNA-regulated network analysis

DNA methylation plays a vital role in gene expression and prognostic assessment. We used GSCALite (http://bioinfo.life.hust.edu.cn/web/GSCALite/) to determine the differential methylation expression and prognostic patterns of COL6A in pan-cancer [25]. In addition, we used TargetScan (http://www.targetscan.org/) to investigate the potential regulatory miRNAs of COL6A, and Cytoscape was used to construct a miRNA-regulated network of COL6A [26].

## Results

### Expression of COL6A in pan-cancer

**Fig. 1:**
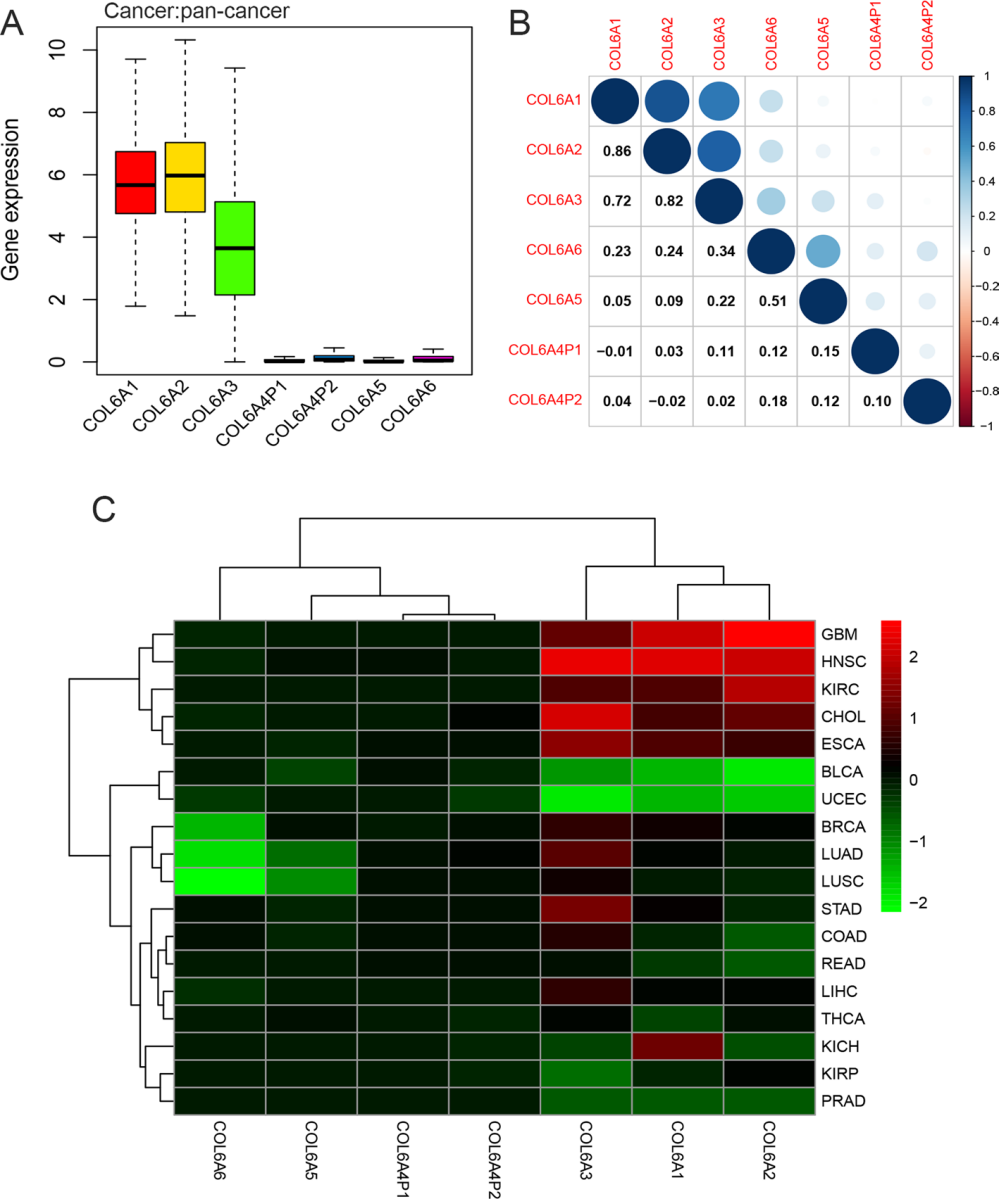
Expression of collagen VI family in pan-cancer. **A** The box plot of the distribution of COL6A gene expression in 33 cancer types. **B** The correlation of COL6A gene expression in 33 cancer types. The blue and red dots indicate a positive and negative relationship, respectively. **C** The heatmap of the differential transcription levels of COL6A members in comparison tumor to adjacent normal tissues based on log2 (fold change) in 18 tumor types with more than five adjacent normal samples. Red represents high expression in tumor tissue and green represents low expression in tumor tissues

Based on published data, a total of seven COL6A members, including COL6A1, COL6A2, COL6A3, COL6A4P1, COL6A4P2, COL6A5, and COL6A6, were analyzed in the present study. To explore the intrinsic expression profiles of COL6A, we first examined gene expression levels in all 33 TCGA tumor tissues. Our results indicated striking inter-cancer heterogeneity in the expression levels of COL6A1/2/3 in pan-cancer. Further analysis found that COL6A1 and COL6A2 were relatively highly expressed in all cancer types compared with other COL6A members. By contrast, COL6A3 was moderately expressed, and COL6A4P1/4P2/5/6 had lower expression levels (Fig. 1A).Using the HPA database, we subsequently tested mRNA expression in normal tissues. The results showed that the mRNA expression levels of COL6A1/2/3 were generally higher in normal tissues compared to COL6A5/6, which were mainly enriched in the lung tissues (Additional file 2). In addition, coexpression analysis revealed significantly positive correlations between COL6A1 and COL6A2 (R = 0.86), followed by COL6A2-COL6A3 (R = 0.82), COL6A1-COL6A3 (R = 0.72), and COL6A5-COL6A6 (R = 0.51) (Fig. 1B).

We also analyzed RNA-seq data from the TCGA database to detect the differential expression of COL6A between tumor tissues and normal tissues in 18 tumors. The fold change (log FC) of COL6A members in different cancer types is summarized in detail in Additional file 1: Table S3. Our results revealed that COL6A was abnormally expressed in a variety of tumor types, either down-expressed or up-expressed in different tumors. For instance, COL6A1 was overexpressed in some specific tumors, including BRCA, CHOL, ESCA, GBM, HNSC, KICH, and KIRC, while the lower expression of COL6A1 was discovered in BLCA, PRAD, THCA, and UCEC. The other COL6A members showed different expression trends in 18 tested cancer types, depending on the specific tumor type. Interestingly, we also noticed that there were significantly opposite expression trends of different COL6A members in the same tumor. For pan-lung: LUAD and LUSC, low expression levels of COL6A5 and COL6A6 were observed, which was opposite to the expression levels of COL6A3, COL6A4P1 and COL6A4P2, and the expression levels of COL6A1 and COL6A2 had no significant changes. The significant overexpression of COL6A5 and downregulation of COL6A6 were observed in BRCA (Fig. 1C).

### Prognostic role of COL6A in pan-cancer

**Fig. 2:**
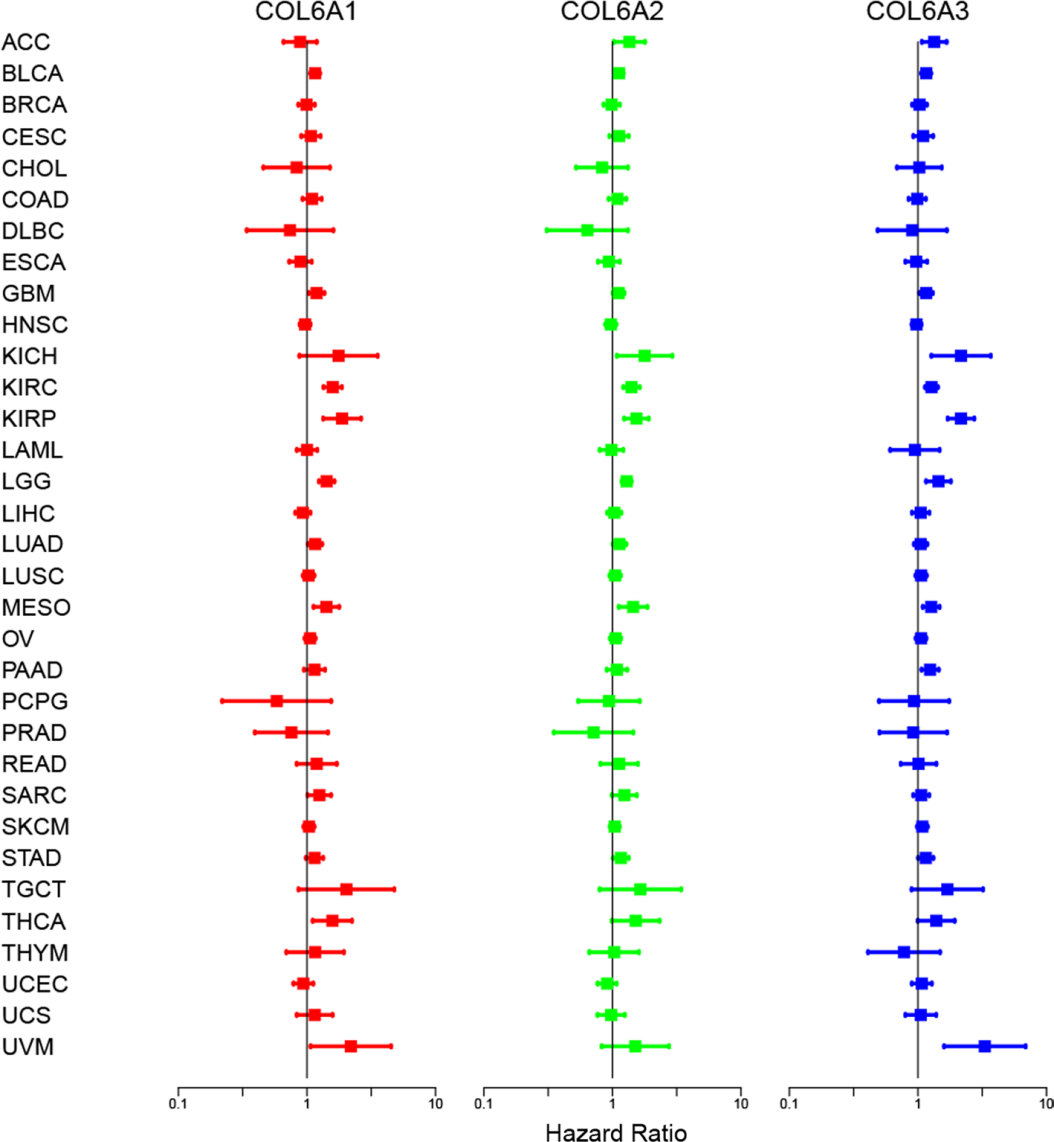
Survival analysis of collagen VI family in pan-cancer. The forest plots show the correlation between COL6A gene expression and overall survival rate by the Cox method. HR < 1 represents low risk, while HR > 1 represents high risk. The details are described in the Additional file 1: Table S4. HR: Hazard ratio

The dysregulated COL6A members were found in pan-cancer, but their prognostic value remained unclear. Therefore, we further explored the association of expression levels of COL6A members with overall survival by Cox regression models, and HR > 1 was considered as a poor prognostic factor. The results revealed that COL6A1/2/3 were prognostic risk factors with HR > 1 in multiple cancer types (Fig. 2 and Additional file 1: Table S4). Specifically, these three genes were predicted poor prognosis in patients with BLCA, GBM, KIRC, KIRP, LGG, and MESO. Notably, COL6A4P1 was associated with lower survival in BRCA, LGG, and LIHC, while it was predicted to have a better prognosis for KICH and PCPG. COL6A5 correlated with poor prognosis of PCPG and UCEC, but favored survival of patients with HNSC and LUAD. In addition, increased COL6A6 expression was predicted poor prognosis for KIRP, READ, KICH, and UCEC, and a better survival rate in LUAD. We further used Kaplan–Meier survival curve to evaluate the prognosis risk of COL6A in 33 cancer types. The results showed that COL6A members were still significantly associated with patients’ overall survival in most tumor types. It is also worth noting that all COL6A members played adverse prognostic roles in patients with KIRC (Additional file 3).

### Association of COL6A with immune subtypes, TME, and stemness scores in pan-cancer

**Fig. 3:**
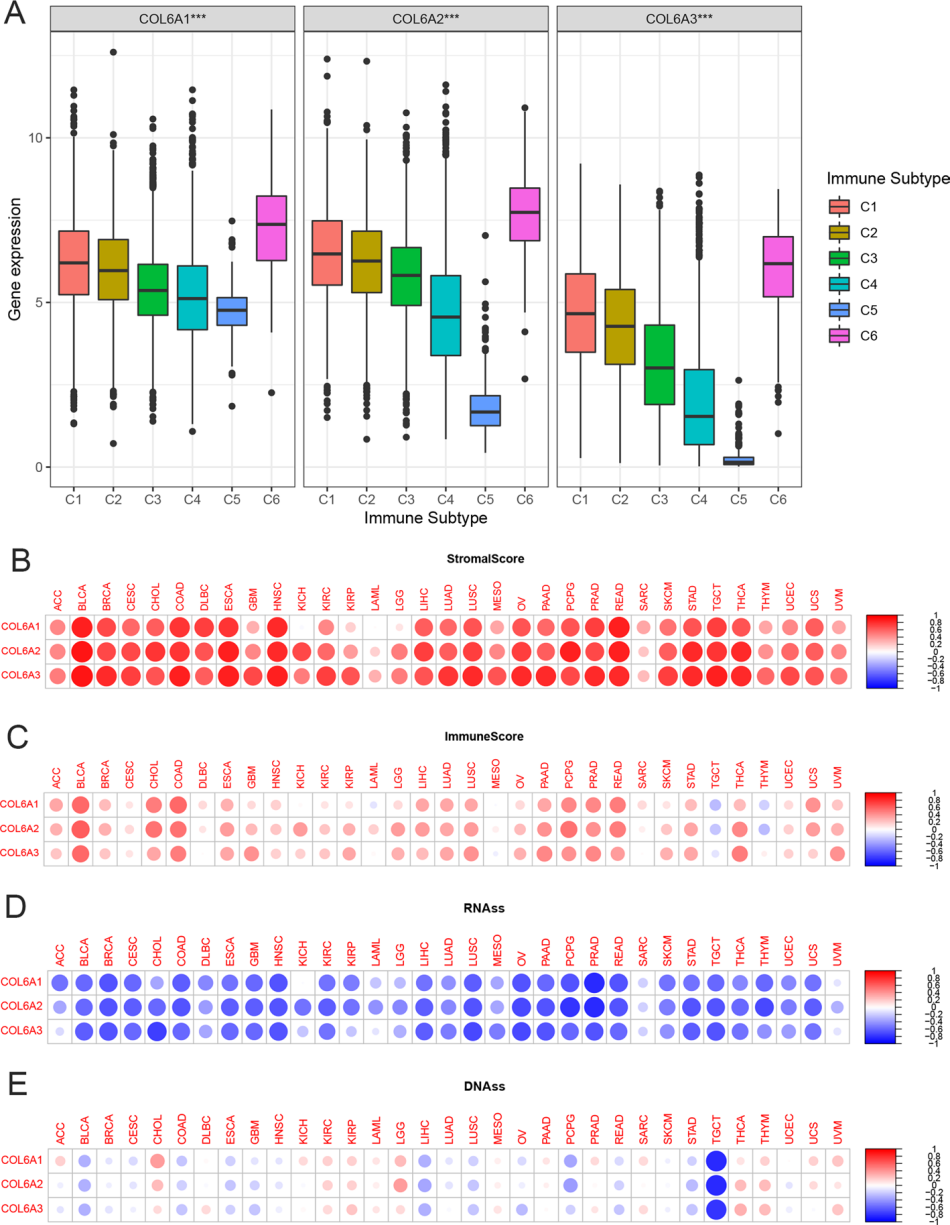
Association of collagen VI Family associated with immune subtypes, tumor microenvironment, and stemness scores in pan-cancer. **A** COL6A gene expression levels in C1-C6 immune subtypes. C1, wound healing; C2, IFN-γ dominant; C3, inflammatory; C4, lymphocyte depleted; C5, immunologically quiet; C6, TGF-β dominant. *p < 0.05; **p < 0.01; ***p < 0.001. **B–C** The two heatmaps of the association of COL6A gene expression with stromal scores and immune scores in different cancers, respectively. **D–E** The two heatmaps of the association of COL6A gene expression with RNAss and DNAss in different cancers, respectively. Red dots represent positive correlation, while blue dots represent negative correlation, and the darker the color, the greater the absolute value of the correlation coefficient. The size of the dots represents statistical significance. RNAss: RNA-based stemness scores; DNAss: DNA methylation-based stemness scores

After that, we focused on three major collagen VI isoforms, including COL6A1, COL6A2, and COL6A3, as a further research object. To understand their association with immune components, we analyzed the expression levels of COL6A1/2/3 in six immune subtypes from 33 tumors. The results indicated that COL6A1/2/3 were associated with immune subtypes (all *p* < 0.001), and had similar expression profiles in six immune subtypes. More specifically, they had the highest expression in C6, followed by C1, C2, and C3, while the lowest expression in C4 and C5 (Fig. 3A). These results suggested that the role of COL6A1/2/3 in inhibiting or promoting cancers may be associated with their immune effects.

We further investigated the relationship between the expression levels of COL6A1/2/3 and infiltrating stromal cells and immune cells in 33 cancers. ESTIMATE program was used to calculate stromal scores and immune scores. The results indicated that COL6A1/2/3 expression had a strong positive association with stromal scores and immune scores in multiple tumor types (Fig. 3B and C), but had a negative correlation with immune scores in TGCT and THCY, indicating that elevated expression levels of three genes were correlated with lower tumor purity.

Furthermore, we explored the relationship between COL6A1/2/3 and stem cell-like characteristics of 33 cancer types based on mRNA expression and stemness scores. The results indicated that COL6A1/2/3 had strong negative correlations with RNAss in most tumors. However, we also noticed that there was no significant association between COL6A1 expression and RNAss in KICH (Fig. 3D). Moreover, we found that there were differences between expression levels of COL6A1/2/3 and DNAss in various cancer types. Specifically, they were negatively associated with DNAss in BLCA, LIHC, and TGCT, while positively correlated with DNAss in CHOL, THCA, and THYM (Fig. 3E).

### Drug sensitivity analysis of COL6A in pan-cancer

**Fig. 4:**
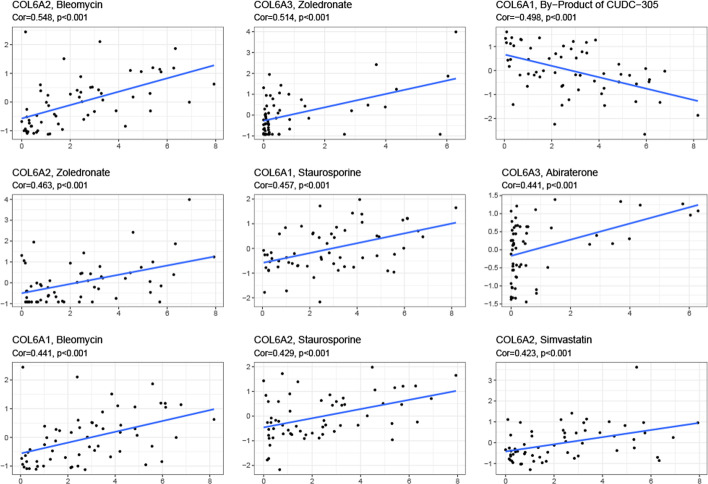
Drug sensitivity analysis of collagen VI family in pan-cancer. The scatter plots of the correlation between drug sensitivity and COL6A1/2/3 in NCI-60 cell lines. The scatter plots are ranked by *p-*value. Cor, correlation coefficient

In order to explore the effects of COL6A1/2/3 on drug treatment, we analyzed their expression in NCI-60 cell lines, and conducted the Pearson’s correlation test to investigate the association between gene expression and drug sensitivity. The results showed that increased expression of COL6A1/2/3 was associated with drug sensitivity of distinct cell lines to multiple chemotherapeutic drugs (Fig. 4 and Additional file 1: Table S5). For instance, COL6A2 was related to cell sensitivity to bleomycin, zoledronate, taurosporine and simvastatin. COL6A3 was positively associated with zoledronate. However, COL6A1/2/3 were also associated with the resistance to several drugs. Moreover, we also noticed that different genes had similar associations with the same drug. For example, COL6A1/2/3 were all positively associated with staurosporine, while negatively correlated with by-products of CUDC-305. These findings indicated that COL6A1/2/3 could serve as potential treatment targets.

### Underlying molecular mechanism analysis of COL6A

**Fig. 5:**
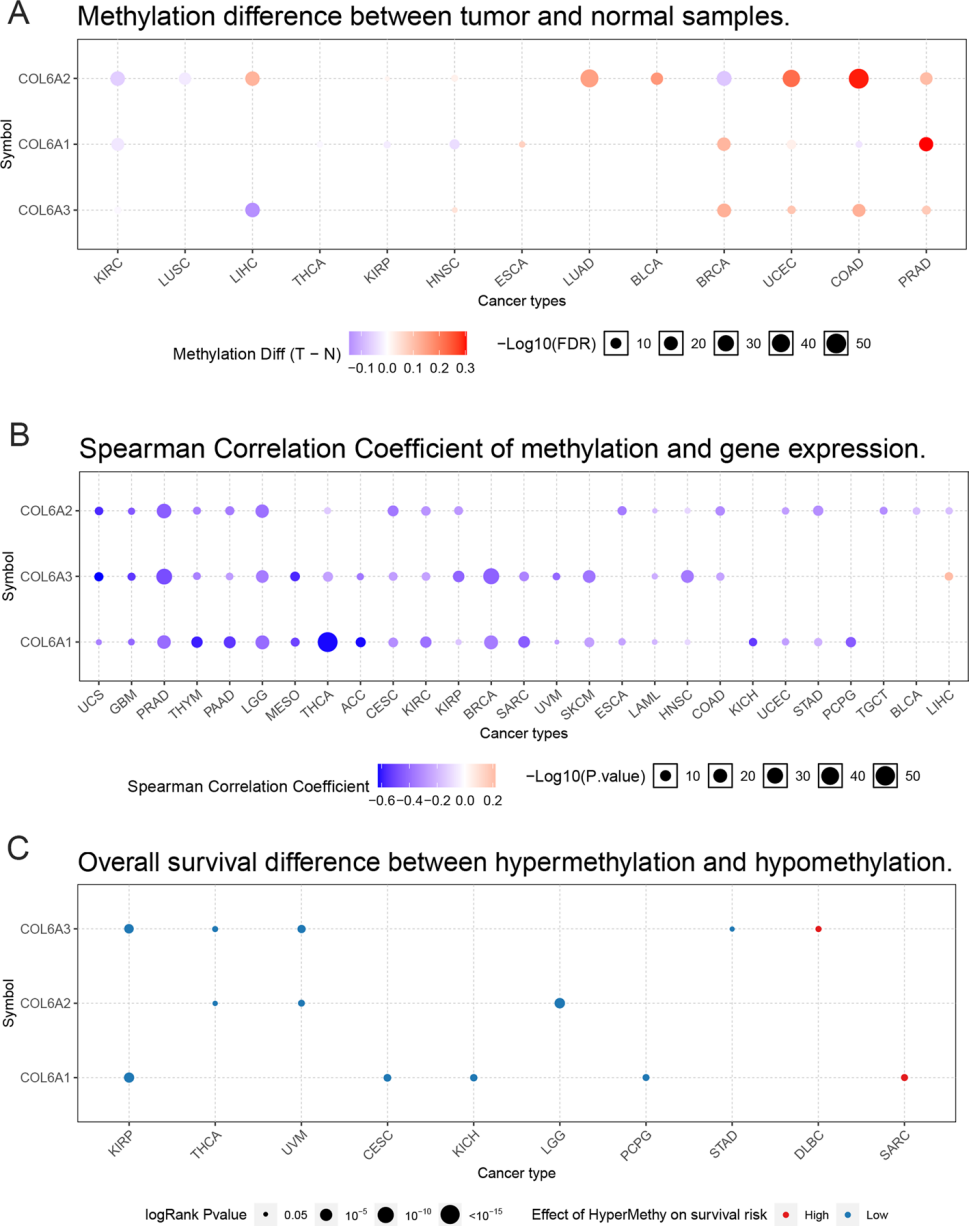
Methylation analysis of collagen VI family in pan-cancer. **A** Differential methylation levels of COL6A1/2/3 between TCGA cancers and adjacent normal tissues. Blue and red dots represent down-regulation and up-regulation of methylation in tumors, and the darker the dots, the greater the differences. The size of the dots indicates statistical significance. **B** Correlation between methylation levels and expression levels of COL6A. Blue and red represent negative correlation and positive correlation, respectively. **C** Effects of hypermethylation of COL6A on the overall survival risk. Red dots represent high risk, while blue dots represent low risk. The size of the dots indicates statistical significance

To further understand the potential molecular mechanism of COL6A1/2/3 in cancers, we first identified the effects of methylation patterns of COL6A1/2/3 in pan-cancer by GSCALite. Our results showed that the methylation levels of COL6A1/2/3 in tumor tissues were significantly lower than in normal tissues, as follows: COL6A1 in ESCA, BRCA, UCEC, and PRAD; COL6A2 in LIHC, HNSC, KIRP, and BLCA; COL6A3 in HNSC, BRCA, UCEC, COAD, and PRAD. However, these genes had higher methylation levels in tumor tissues, including COL6A1 in KIRC, HNSC, THCA, KIRP, and COAD; COL6A2 in KIRC, LUSC, and BRCA; COL6A3 in LIHC and KIRC (Fig. 5A). In addition, the expression of COL6A1/2/3 was mainly negatively correlated with methylation, except for COL6A3 in LIHC (Fig. 5B). Survival analyses revealed that the hypermethylation levels of COL6A1/2/3 were risk factors to predict prognosis in most cancer types, but the hypermethylation level was identified as a better prognostic factor for COL6A3 in DLBC and COL6A1 in SARC (Fig. 5C).We further constructed the miRNA-to-gene network of COL6A1/2/3. Our results showed that COL6A members were regulated by more than one miRNA. To be specific, COL6A1 was regulated by 67 miRNAs, COL6A2 was regulated by 21 miRNAs, while COL6A3 was regulated by 67 miRNAs. In addition, we also observed that the same miRNA could regulate multiple genes, such as hsa-miR-29-3p, which regulated COL6A1 and COL6A2 (Additional file 4).

### Role of COL6A in colorectal cancer

**Fig. 6:**
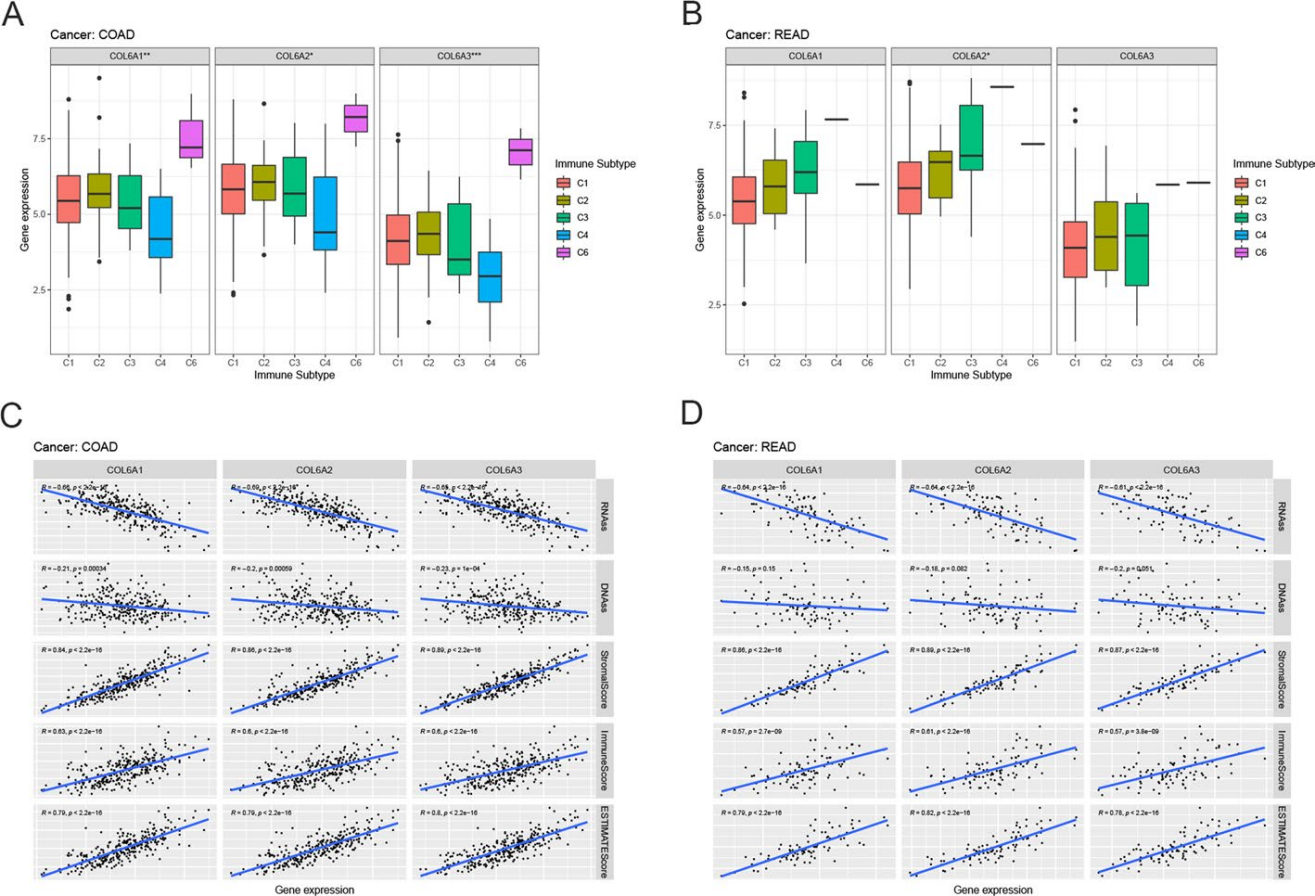
The role of collagen VI family in colorectal cancer. **A**–**B** COL6A gene expression levels in C1-C6 immune subtype in COAD and READ, respectively. C1, wound healing; C2, IFN-γ dominant; C3, inflammatory; C4, lymphocyte depleted; C5, immunologically quiet; C6, TGF-β dominant. *p < 0.05; **p < 0.01; ***p < 0.001. **C–D** The correlation between COL6A gene expression and RNAss, DNAss, stromal scores, immune score, and estimate scores in COAD and READ, respectively. R means correlation coefficient. P represents statistical significance. RNAss: RNA-based stemness scores; DNAss: DNA methylation-based stemness scores; COAD: Colon adenocarcinoma; READ: Rectum adenocarcinoma

Considering the heterogeneity of tumors from the same origin, we further investigated the role of COL6A1/2/3 in COAD and READ. Based on the immunohistochemical results from the HPA database, we studied the protein expression of COL6A1/2/3 in colorectal cancer tissues (Additional file 5). Besides, the CCLE database was used to show the expression of COL6A1/2/3 in colorectal cancer cell lines (Additional file 5). The expression patterns of COL6A1/2/3 were similar in six immune subtypes of COAD. More specifically, COL6A1/2/3 expression was relatively high in C6 while low in C4 (Fig. 6A). COL6A2 expression varied in six immune subtypes of READ and was higher in C4 (p < 0.05) (Fig. 6B). Very interestingly, there was no C5 subtype of COAD and READ. Figure 6C shows that the expression of COL6A1/2/3 in patients with COAD was significantly negatively associated with the RNAss and DNAss (*p* < 0.05), while positively correlated with stromal scores, immune scores, and estimate scores, and similar results were observed in READ (Fig. 6D).

## Discussion

In this study, we conducted a systematic and comprehensive description analysis of the features of COL6A. We first performed intrinsic expression analysis, and our results demonstrated that the gene expression levels of COL6A1/2/3 were relatively higher than 2, whereas the expression levels of other COL6A members (COL6A4/5/6) were less than 1 in pan-cancer, indicating that the expression levels among different COL6A members showed a remarkably heterogeneous distribution. The reason for the relatively lower expression of COL6A4/5/6 chains compared to regular chains may be related to a large pericentric inversion on chromosome 3, leading to inactivation of COL6A4 and inhibition of COL6A5/6 transcription [7]. We further conducted coexpression analysis of COL6A members in pan-cancer, and found that COL6A1, COL6A2 and COL6A3 were highly positively correlated. Indeed, previous research has shown that collagen VI assembles into a triple-helical monomer made up of three main chains (α1, α2 and α3) with a 1:1:1 stoichiometric ratio [8]. Therefore, these results suggested that COL6A1/2/3 might work together and share some common functions. Unexpectedly, our study also revealed the great heterogeneity in the expression of COL6A members in the same tumor. Consistent with the above findings, previous studies have reported that COL6A1 and COL6A2 expression levels are downregulated in both non-muscle invasive bladder cancer (NMIBC) and MIBC tissue samples [15]. In contrast, COL6A3 has been proved to be highly expressed in bladder cancer tissues and cells [27]. Moreover, we confirmed that the same COL6A member had different expression levels in different cancers, even in tumor with the similar tissue origin. Based on previous studies and our findings, this may be due to the restricted tissue distribution of COL6A [7], and COL6A4/5/6 share homology with the COL6A3 chain, as these chains are often expressed complementarily on certain basement membranes [6]. In addition, in this study, aberrant methylation of COL6A1/2/3 was observed in some cancers, suggesting that abnormal expression of COL6A1/2/3 may be regulated by methylation modification in specific tumors. However, our findings were obtained from bioinformatics analysis based on public databases, and more laboratory studies are needed to confirm our conclusions in the future.

Another essential finding has suggested that COL6A gene expression levels were correlated with immune subtypes in pan-cancer, and COL6A1/2/3 all have the highest expression in the C6 immune subtype, which has the worst prognosis among the six immune subtypes [21]. This may explain the above finding that COL6A1/2/3 were mainly correlated with poor overall survival outcomes. Previously, high expression of COL6A1 has been reported to predict poor prognosis in pancreatic cancer and cervical cancer [13, 28], and similar effects of COL6A1/2/3 are also seen in triple-negative breast cancer (TNBC) [29]. Taken together, COL6A1/2/3 may become potential prognostic markers in clinical application.

The investigation into the interactions with tumor cells, immune cells, and stromal cells in TME is extremely essential for tumoreigenesis and provided new perceptions on exploring more powerful treatment mean. Thus, based on the ESTIMATE algorithm, we further calculated stromal scores and immune scores of 33 TCGA cancer types. Our study found that COL6A1/2/3 were positively associated with the infiltration levels of stromal cells and immune cells. Similar to our results, recent studies have indicated that COL6A is abundantly expressed and secreted by primary macrophages and macrophage cell lines, which is in return to modulate cell–matrix and cell–cell interactions[30]. COL6A1 is packaged into osteosarcoma cell-derived exosomes and activates cancer-associated fibroblasts in TME [11]. Therefore, COL6A may be an important linkage between malignant cells and TME.

Stemness scores have been proposed to describe the self-renewal and dedifferentiation of stem cell-like characteristics. Cancer stem cells have been reported to be influenced in many tumor progressions related to tumorigenesis, distant metastasis, and chemotherapy resistance. Besides, the acquisition of stem cell-like properties correlates with the biological and molecular heterogeneity of cancer [23, 31]. In the present study, the correlation of COL6A1/2/3 with tumor stemness scores was explored. We found that COL6A1/2/3 were negatively associated with RNAss within tumors. These findings have been partially proved in previous studies. For example, Chih-Ming Ho et al. have reported that collagen VI promotes ovarian cancer cell stemness by regulating the CDK4/6-p-Rb signaling pathway [32]. Endotrophin (ETP), a cleavage product of the COL6A3 chain, increases tumor stem cell-like cells though activating the anthrax toxin receptor 1 (ANTXR1). Thus, the interactions of ANTXR with ETP make a bridge of a network of collagen cleavage and remodeling in TME [33]. COL6A1 mediates Fzd7-Wnt5b to induce breast cancer mesenchymal-like stemness [34]. As a result, COL6A may play a pivotal role in tumor-initiating cells. It is also worth noting that the expression of COL6A1/2/3 is positively or negatively associated with the sensitivity to specific drugs. Previous studies have shown that overexpression of COL6A3 promotes cisplatin resistance in ovarian cancer cells and breast cancer cells [35, 36]. COL6A3 has also been reported to be related to oxaliplatin resistance in ovarian cancer cells [37]. Taken together, previous studies and our findings all suggest that COL6A can be used as potential therapeutic targets for antitumor treatment.

## Conclusion

This research showed the similarities and differences of molecular characteristic in collagen VI family in pan-cancer, especially COL6A1/2/3. In summary, our results indicated that COL6A members gene expression levels showed great heterogeneity, which needs to be studied in specific cancer types in the future. Moreover, the expression levels of COL6A1, COL6A2, and COL6A3 showed significantly positive correlation, and had similar effects on the prognostic value, immune subtypes, TME, and RNAss. Overall, our work revealed their roles in expression, prognosis, DNA methylation, immune reaction, TME, tumor stemness and drug sensitivity. This study will verify the pre-existing hypotheses, and offer new clues for exploring potential mechanism of collagen VI family in 33 cancer types.

## Supplementary Information

**Additional file 1.** Supplementary tables.

**Additional file 2.** Collagen VI family expression in normal tissues based on the HPA database.

**Additional file 3.** Kaplan-Meier plots showing the association between collagen VI family gene expression and overall survival in KIRC.

**Additional file 4.** The miRNA-regulated network of collagen VI family.

**Additional file 5.** Expression of collagen VI family in colorectal cancer.

## Data Availability

The datasets analyzed during the current study are available in the UCSC Xena database (http://xena.ucsc.edu/), CellMiner database (https://discover.nci.nih.gov/cellminer/), Human Protein Atlas database (https://www.proteinatlas.org/), Cancer Cell Line Encyclopedia database (https://portals.broadinstitute.org/ccle), GSCALite (http://bioinfo.life.hust.edu.cn/web/GSCALite/), and TargetScan (http://www.targetscan.org/).

## Abbreviations

COL6A: Collagen VI family
ECM: Extracellular matrix
TCGA: The Cancer Genome Atlas
TME: Tumor microenvironment
HR: Hazard ratio
OCLR: One-class logistic regression
RNAss: RNA-based stemness scores
DNAss: DNA methylation-based stemness scores
NMIBC: Non-muscle invasive bladder cancer
