# Supplementary material for: A pan-cancer analysis of collagen VI family on prognosis, tumor microenvironment, and its potential therapeutic effect
Source: BMC Bioinformatics. 2022 Sep 27;23:390. doi: 10.1186/s12859-022-04951-0 (PMC9513866; doi:10.1186/s12859-022-04951-0)
Supplement: Supplementary file 1 — Additional file 1. Supplementary tables. [file 12859_2022_4951_MOESM1_ESM.docx]

**Table S1. Basic information of 33 tumor types from TCGA.**

| Tumor type | Tumor sample (N) | Normal sample (N) |
| --- | --- | --- |
| Adrenocortical carcinoma (ACC) | 79 | 0 |
| Bladder urothelial carcinoma (BLCA) | 411 | 19 |
| Breast carcinoma (BRCA) | 1118 | 99 |
| Cervical squamous cell carcinoma and endocervical adenocarcinoma (CESC) | 306 | 3 |
| Cholangiocarcinoma (CHOL) | 36 | 9 |
| Colon adenocarcinoma (COAD) | 471 | 41 |
| Lymphoid neoplasm diffuse large b-cell lymphoma (DLBC) | 48 | 0 |
| Esophageal carcinoma (ESCA) | 162 | 11 |
| Glioblastoma multiforme (GBM) | 168 | 5 |
| Head and neck squamous cell carcinoma (HNSC) | 502 | 44 |
| Kidney chromophobe (KICH) | 65 | 24 |
| Kidney renal clear cell carcinoma (KIRC) | 535 | 72 |
| Kidney renal papillary cell carcinoma (KIRP) | 289 | 32 |
| Acute myeloid leukemia (LAML) | 151 | 0 |
| Brain lower-grade glioma (LGG) | 529 | 0 |
| Liver hepatocellular carcinoma (LIHC) | 374 | 50 |
| Lung adenocarcinoma (LUAD) | 527 | 58 |
| Lung squamous cell carcinoma (LUSC) | 501 | 49 |
| Mesothelioma (MESO) | 86 | 0 |
| Ovarian serous cystadenocarcinoma (OV) | 379 | 0 |
| Pancreatic adenocarcinoma (PAAD) | 178 | 4 |
| Pheochromocytoma and paraganglioma (PCPG) | 183 | 3 |
| Prostate adenocarcinoma (PRAD) | 500 | 51 |
| Rectum adenocarcinoma (PEAD) | 167 | 10 |
| Sarcoma (SARC) | 263 | 2 |
| Skin cutaneous melanoma (SKCM) | 471 | 1 |
| Stomach adenocarcinoma (STAD) | 375 | 32 |
| Testicular germ cell tumors (TGCT) | 156 | 0 |
| Thyroid carcinoma (THCA) | 512 | 56 |
| Thymoma (THYM) | 119 | 2 |
| Uterine corpus endometrial carcinoma (UCEC) | 548 | 35 |
| Uterine carcinosarcoma (UCS) | 56 | 0 |
| Uveal melanoma (UVM) | 80 | 0 |

**Table S2. The information of** **collagen VI family.**

| Gene ID | Symbol | Description |
| --- | --- | --- |
| 1291 | COL6A1 | collagen type VI alpha 1 chain |
| 1292 | COL6A2 | collagen type VI alpha 2 chain |
| 1293 | COL6A3 | collagen type VI alpha 3 chain |
| 344875 | COL6A4P1 | collagen type VI alpha 4 pseudogene 1 |
| 646300 | COL6A4P2 | collagen type VI alpha 4 pseudogene 2 |
| 256076 | COL6A5 | collagen type VI alpha 5 chain |
| 131873 | COL6A6 | collagen type VI alpha 6 chain |

**Table S3. Log2 (fold change) of collagen VI family in comparing tumor to adjacent normal tissue across 18 cancer types.**

| Cancer Type | COL6A1 | COL6A2 | COL6A3 | COL6A4P1 | COL6A4P2 | COL6A5 | COL6A6 |
| --- | --- | --- | --- | --- | --- | --- | --- |
| BLCA | -1.39557 | -1.87446 | -1.18332 | 0.078347 | -0.0877 | -0.34849 | 0.016116 |
| BRCA | 0.354044 | 0.195507 | 0.648116 | 0.027717 | 0.042355 | 0.032328 | -1.39314 |
| CHOL | 0.879953 | 1.106068 | 2.140799 | 0.009227 | 0.158195 | 0.016247 | -0.15131 |
| COAD | -0.13977 | -0.56345 | 0.567416 | 0.067871 | 0.061156 | -0.13987 | 0.048115 |
| ESCA | 0.891462 | 0.748013 | 1.496695 | 0.056232 | 0.061311 | -0.13405 | -0.06245 |
| GBM | 2.100287 | 2.594638 | 1.125982 | 0.002239 | -0.03247 | 0.011358 | -0.07551 |
| HNSC | 2.239415 | 2.047979 | 2.368488 | 0.0589 | 0.029248 | 0.034445 | -0.07028 |
| KICH | 1.202063 | -0.49605 | -0.3575 | -0.0569 | -0.08961 | 0.001567 | -0.0064 |
| KIRC | 0.939767 | 1.859346 | 0.934349 | -0.02621 | -0.00956 | -0.02489 | -0.02192 |
| KIRP | -0.08914 | 0.136804 | -0.77806 | -0.02443 | -0.12298 | -0.01629 | -0.03334 |
| LIHC | 0.135462 | 0.138953 | 0.625121 | 0.022392 | 0.027809 | 0.003644 | -0.15995 |
| LUAD | 0.166581 | -0.06308 | 1.044208 | 0.088877 | 0.185852 | -0.74711 | -1.82975 |
| LUSC | 0.023654 | -0.14047 | 0.345888 | 0.123845 | 0.090427 | -1.01432 | -2.15139 |
| PRAD | -0.62297 | -0.54175 | -0.60439 | 0.006811 | 0.004711 | -0.02166 | 0.001614 |
| READ | -0.33283 | -0.60843 | 0.112231 | 0.062492 | 0.076991 | -0.0325 | 0.007577 |
| STAD | 0.283541 | -0.06426 | 1.32629 | 0.0876 | 0.085834 | -0.09371 | 0.059182 |
| THCA | -0.38435 | 0.110703 | 0.208963 | -0.02391 | -0.06732 | 0.063775 | -0.06261 |
| UCEC | -1.41332 | -1.64687 | -1.94956 | -0.04262 | -0.28096 | 0.014401 | -0.32274 |

**Table S4. Association of collagen VI family with the patient overall survival in different cancers by COX analysis.**

| Gene | Cancer | HR | HR.95L | HR.95H | *p*-value |
| --- | --- | --- | --- | --- | --- |
| COL6A1 | BLCA | 1.15744 | 1.060947 | 1.262709 | 0.000995 |
|  | GBM | 1.188516 | 1.03355 | 1.366716 | 0.015396 |
|  | KIRC | 1.585998 | 1.3462 | 1.868512 | 3.50E-08 |
|  | KIRP | 1.878222 | 1.337256 | 2.638027 | 0.000276 |
|  | LGG | 1.423649 | 1.238524 | 1.636444 | 6.70E-07 |
|  | LUAD | 1.156261 | 1.0183 | 1.312914 | 0.02511 |
|  | MESO | 1.415743 | 1.125289 | 1.781169 | 0.003002 |
|  | SARC | 1.247914 | 1.010362 | 1.541317 | 0.039816 |
|  | THCA | 1.577218 | 1.109209 | 2.242693 | 0.011179 |
|  | UVM | 2.194159 | 1.065709 | 4.517494 | 0.032951 |
| COL6A2 | ACC | 1.356066 | 1.026218 | 1.791935 | 0.032196 |
|  | BLCA | 1.125549 | 1.037384 | 1.221207 | 0.004485 |
|  | GBM | 1.117313 | 1.005899 | 1.241068 | 0.03848 |
|  | KICH | 1.779976 | 1.081647 | 2.929156 | 0.023281 |
|  | KIRC | 1.40718 | 1.214043 | 1.631044 | 5.76E-06 |
|  | KIRP | 1.536786 | 1.233722 | 1.914299 | 0.000126 |
|  | LGG | 1.288941 | 1.180362 | 1.407508 | 1.57E-08 |
|  | LUAD | 1.134473 | 1.00467 | 1.281046 | 0.041839 |
|  | MESO | 1.447813 | 1.120445 | 1.87083 | 0.004661 |
|  | STAD | 1.16335 | 1.009661 | 1.340433 | 0.036352 |
| COL6A3 | ACC | 1.339106 | 1.072426 | 1.672103 | 0.009964 |
|  | BLCA | 1.159134 | 1.060938 | 1.266419 | 0.001077 |
|  | GBM | 1.157888 | 1.026372 | 1.306256 | 0.017166 |
|  | KICH | 2.165132 | 1.271888 | 3.685699 | 0.004427 |
|  | KIRC | 1.274021 | 1.134987 | 1.430086 | 4.00E-05 |
|  | KIRP | 2.162604 | 1.703062 | 2.746145 | 2.48E-10 |
|  | LGG | 1.444501 | 1.156042 | 1.804938 | 0.001213 |
|  | MESO | 1.268559 | 1.091295 | 1.474618 | 0.001951 |
|  | PAAD | 1.243804 | 1.065547 | 1.451883 | 0.005703 |
|  | STAD | 1.150413 | 1.003851 | 1.318373 | 0.043879 |
|  | UVM | 3.312307 | 1.594532 | 6.880624 | 0.001323 |
| COL6A4P1 | BRCA | 3.878472 | 1.114046 | 13.50263 | 0.033201 |
|  | KICH | 6.99E+50 | 4.83E+16 | 1.01E+85 | 0.003532 |
|  | LGG | 8.009104 | 1.9172 | 33.45803 | 0.004341 |
|  | LIHC | 777.1557 | 17.25577 | 35001.11 | 0.000612 |
|  | PCPG | 3.84E+10 | 103450.2 | 1.43E+16 | 0.000196 |
| COL6A5 | HNSC | 0.328603 | 0.118316 | 0.912641 | 0.032732 |
|  | LUAD | 0.587893 | 0.405937 | 0.851408 | 0.004934 |
|  | PCPG | 6.180729 | 2.138628 | 17.86258 | 0.000769 |
|  | UCEC | 19.25046 | 2.937884 | 126.1384 | 0.002045 |
| COL6A6 | KIRP | 1894.855 | 28.90843 | 124201.7 | 0.000406 |
|  | READ | 39.6461 | 3.337036 | 471.0207 | 0.003565 |
|  | LUAD | 0.684481 | 0.530016 | 0.883963 | 0.003671 |
|  | UVM | 9.15E-17 | 1.45E-28 | 5.78E-05 | 0.007726 |
|  | KICH | 671897.2 | 16.58714 | 2.72E+10 | 0.013181 |
|  | UCEC | 2.589495 | 1.21213 | 5.531982 | 0.014022 |

**Table S5. The correlation between COL6A1/2/3 expression levels and drug sensitivity in NCI-60 cell lines.**

| Gene | Drug | Correlation coefficient | *p*-value |
| --- | --- | --- | --- |
| COL6A2 | Bleomycin | 0.547889 | 5.87E-06 |
| COL6A3 | Zoledronate | 0.513787 | 2.69E-05 |
| COL6A1 | By-Product of CUDC-305 | -0.498 | 5.15E-05 |
| COL6A2 | Zoledronate | 0.462939 | 0.000196 |
| COL6A1 | Staurosporine | 0.456964 | 0.000242 |
| COL6A3 | Abiraterone | 0.441323 | 0.000416 |
| COL6A1 | Bleomycin | 0.4408 | 0.000424 |
| COL6A2 | Staurosporine | 0.429295 | 0.00062 |
| COL6A2 | Simvastatin | 0.422687 | 0.000767 |
| COL6A1 | Simvastatin | 0.421234 | 0.000803 |
| COL6A2 | By-Product of CUDC-305 | -0.41881 | 0.000867 |
| COL6A3 | Staurosporine | 0.418618 | 0.000873 |
| COL6A3 | Bleomycin | 0.379878 | 0.002756 |
| COL6A2 | Nilotinib | -0.37777 | 0.002923 |
| COL6A2 | Rapamycin | 0.375431 | 0.003118 |
| COL6A1 | Midostaurin | 0.37082 | 0.003538 |
| COL6A3 | Streptozocin | 0.36865 | 0.003752 |
| COL6A1 | Zoledronate | 0.364331 | 0.004212 |
| COL6A2 | LY-294002 | 0.360815 | 0.004624 |
| COL6A2 | Cobimetinib (isomer 1) | -0.3557 | 0.005284 |
| COL6A2 | Mitoxantrone | 0.353648 | 0.005573 |
| COL6A1 | Irofulven | 0.352931 | 0.005677 |
| COL6A1 | Nilotinib | -0.35255 | 0.005732 |
| COL6A3 | Cobimetinib (isomer 1) | -0.34932 | 0.006225 |
| COL6A2 | Midostaurin | 0.34288 | 0.007321 |
| COL6A1 | Olaparib | 0.337046 | 0.008454 |
| COL6A3 | Dasatinib | 0.333778 | 0.009154 |
| COL6A2 | AFP464 | -0.32822 | 0.01046 |
| COL6A1 | Cyclophosphamide | -0.32797 | 0.010521 |
| COL6A3 | Everolimus | 0.327937 | 0.01053 |
| COL6A3 | Rapamycin | 0.327919 | 0.010535 |
| COL6A2 | Palbociclib | -0.32782 | 0.010559 |
| COL6A2 | Abiraterone | 0.327567 | 0.010623 |
| COL6A3 | Cabozantinib | 0.327388 | 0.010668 |
| COL6A1 | Palbociclib | -0.32691 | 0.01079 |
| COL6A2 | Olaparib | 0.326795 | 0.010819 |
| COL6A3 | Wortmannin | 0.326492 | 0.010897 |
| COL6A3 | By-Product of CUDC-305 | -0.31799 | 0.013289 |
| COL6A2 | Dasatinib | 0.317721 | 0.01337 |
| COL6A2 | Gemcitabine | 0.314322 | 0.014451 |
| COL6A3 | Midostaurin | 0.311749 | 0.015318 |
| COL6A2 | Floxuridine | 0.307314 | 0.016918 |
| COL6A2 | Topotecan | 0.307112 | 0.016994 |
| COL6A1 | Everolimus | 0.307091 | 0.017002 |
| COL6A2 | Selumetinib | -0.30661 | 0.017185 |
| COL6A1 | Bafetinib | -0.30589 | 0.017463 |
| COL6A1 | Amonafide | -0.30526 | 0.017706 |
| COL6A3 | LY-294002 | 0.301653 | 0.019166 |
| COL6A2 | Everolimus | 0.30061 | 0.019607 |
| COL6A3 | Idelalisib | 0.298991 | 0.020308 |
| COL6A3 | 8-Chloro-adenosine | -0.29609 | 0.021617 |
| COL6A3 | Itraconazole | 0.292694 | 0.02324 |
| COL6A3 | Temsirolimus | 0.292204 | 0.023483 |
| COL6A2 | Trametinib | -0.29213 | 0.02352 |
| COL6A2 | Irofulven | 0.29099 | 0.024093 |
| COL6A3 | Trametinib | -0.29024 | 0.024476 |
| COL6A1 | Dolastatin 10 | -0.29023 | 0.024481 |
| COL6A2 | Temsirolimus | 0.28985 | 0.024678 |
| COL6A3 | Mitoxantrone | 0.289632 | 0.024791 |
| COL6A2 | Triapine | 0.289483 | 0.024869 |
| COL6A2 | Bafetinib | -0.28946 | 0.024881 |
| COL6A1 | Rapamycin | 0.286346 | 0.026552 |
| COL6A1 | Hypothemycin | -0.28425 | 0.027727 |
| COL6A1 | Raloxifene | -0.28377 | 0.028006 |
| COL6A1 | LDK-378 | -0.28305 | 0.028421 |
| COL6A2 | Cabozantinib | 0.282861 | 0.028533 |
| COL6A3 | Entinostat | -0.28021 | 0.030124 |
| COL6A1 | Wortmannin | 0.280005 | 0.030248 |
| COL6A2 | LDK-378 | -0.27797 | 0.031518 |
| COL6A2 | Irinotecan | 0.275899 | 0.032863 |
| COL6A2 | Lenvatinib | 0.270949 | 0.036264 |
| COL6A1 | Oxaliplatin | -0.26989 | 0.037025 |
| COL6A3 | Cladribine | -0.26829 | 0.038209 |
| COL6A2 | Itraconazole | 0.266017 | 0.039938 |
| COL6A1 | Dexrazoxane | -0.26479 | 0.040896 |
| COL6A2 | Cyclophosphamide | -0.26306 | 0.042288 |
| COL6A2 | 5-fluoro deoxy uridine 10mer | 0.262733 | 0.042551 |
| COL6A2 | Cisplatin | 0.262553 | 0.042698 |
| COL6A2 | Entinostat | -0.26163 | 0.043459 |
| COL6A2 | Lapatinib | -0.26149 | 0.043578 |
| COL6A1 | LY-294002 | 0.261238 | 0.043786 |
| COL6A2 | 7-Ethyl-10-hydroxycamptothecin | 0.260311 | 0.044567 |
| COL6A2 | AT-13387 | -0.25965 | 0.045132 |
| COL6A1 | Cabozantinib | 0.257789 | 0.046749 |
| COL6A2 | Wortmannin | 0.257543 | 0.046966 |
| COL6A1 | Procarbazine | 0.25551 | 0.048795 |
| COL6A1 | Lapatinib | -0.25507 | 0.049194 |
| COL6A1 | Imexon | -0.25502 | 0.049249 |
| COL6A2 | Idelalisib | 0.25445 | 0.049772 |
