# Supplementary figures and images for: A pan-cancer analysis of collagen VI family on prognosis, tumor microenvironment, and its potential therapeutic effect
Source: BMC Bioinformatics. 2022 Sep 27;23:390. doi: 10.1186/s12859-022-04951-0 (PMC9513866; doi:10.1186/s12859-022-04951-0)

**Additional file 2.** Collagen VI family expression in normal tissues based on the HPA database.

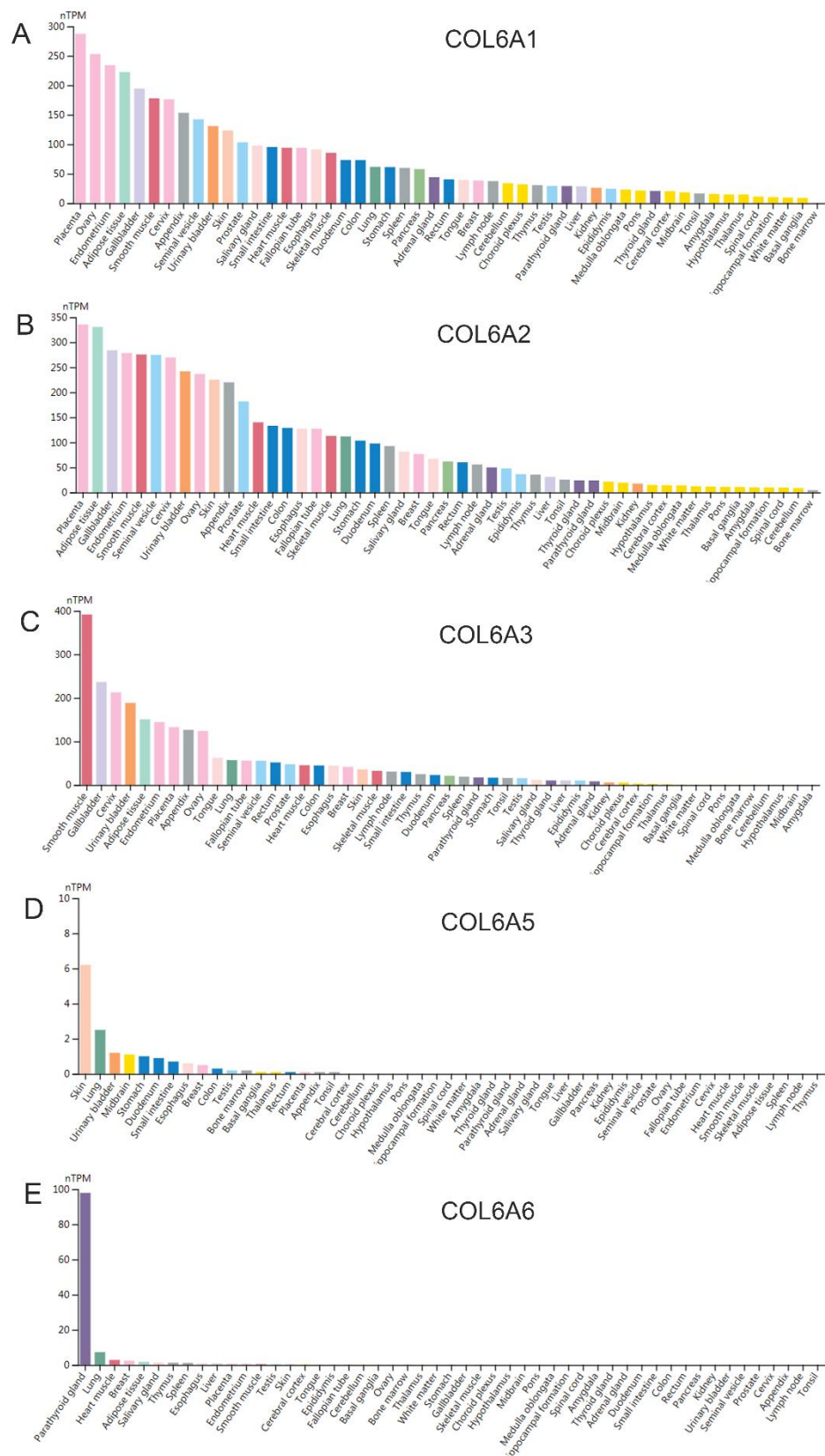

Supplement: Supplementary file 2 — Additional file 2. Collagen VI family expression in normal tissues based on the HPA database. [file 12859_2022_4951_MOESM2_ESM.pdf]
