# Supplementary material for: A pan-cancer analysis of collagen VI family on prognosis, tumor microenvironment, and its potential therapeutic effect
Source: BMC Bioinformatics. 2022 Sep 27;23:390. doi: 10.1186/s12859-022-04951-0 (PMC9513866; doi:10.1186/s12859-022-04951-0)

Additional file 3. Kaplan-Meier plots showing the association between collagen VI family gene expression and overall survival in KIRC. Significance indicated by log-rank tests. Red lines indicate high expression and blue lines indicate low expression. KIRC, kidney renal clear cell carcinoma.

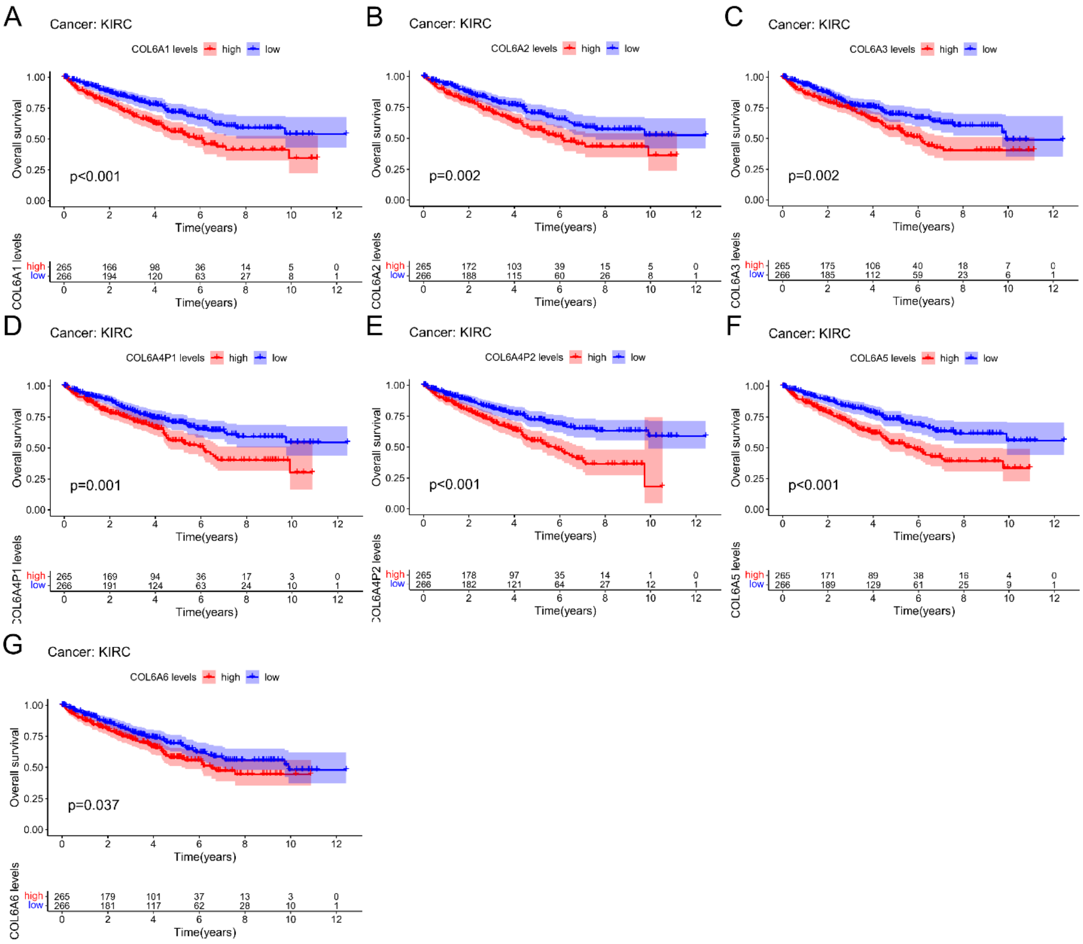

Supplement: Supplementary file 3 — Additional file 3. Kaplan-Meier plots showing the association between collagen VI family gene expression and overall survival in KIRC. [file 12859_2022_4951_MOESM3_ESM.pdf]
