# Supplementary material for: A pan-cancer analysis of collagen VI family on prognosis, tumor microenvironment, and its potential therapeutic effect
Source: BMC Bioinformatics. 2022 Sep 27;23:390. doi: 10.1186/s12859-022-04951-0 (PMC9513866; doi:10.1186/s12859-022-04951-0)

Additional file 4. The miRNA-regulated network of collagen VI family. Pink nodes represent target genes, and green nodes represent miRNAs.

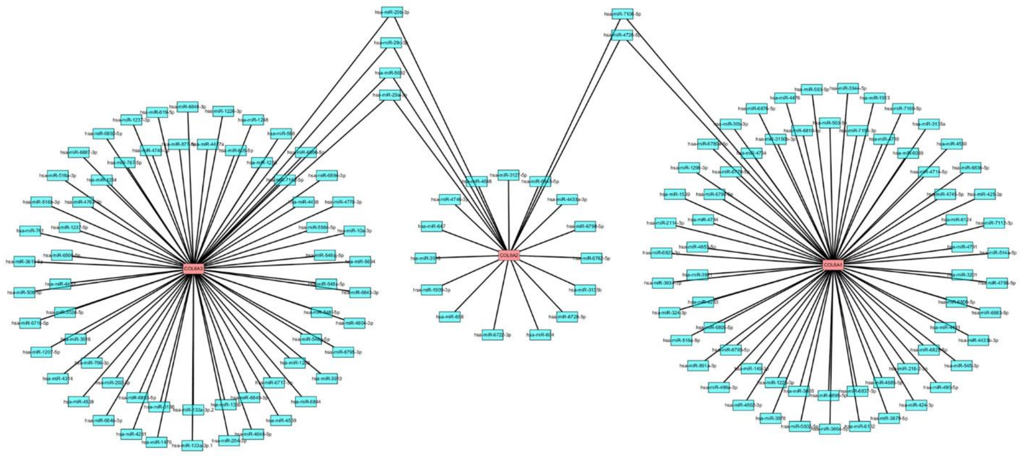

Supplement: Supplementary file 4 — Additional file 4. The miRNA-regulated network of collagen VI family. [file 12859_2022_4951_MOESM4_ESM.pdf]
