# Supplementary material for: A pan-cancer analysis of collagen VI family on prognosis, tumor microenvironment, and its potential therapeutic effect
Source: BMC Bioinformatics. 2022 Sep 27;23:390. doi: 10.1186/s12859-022-04951-0 (PMC9513866; doi:10.1186/s12859-022-04951-0)

Additional file 5. Expression of collagen VI family in colorectal cancer. (A) The protein expression of COL6A1/2/3 in colorectal cancer tissues from HPA database. (B) Heatmap of expression levels of COL6A1/2/3 in colorectal cancer cell lines from the CCLE database. Red indicates over-expression, whereas blue indicates under-expression. CCLE: Cancer Cell Line Encyclopedia.

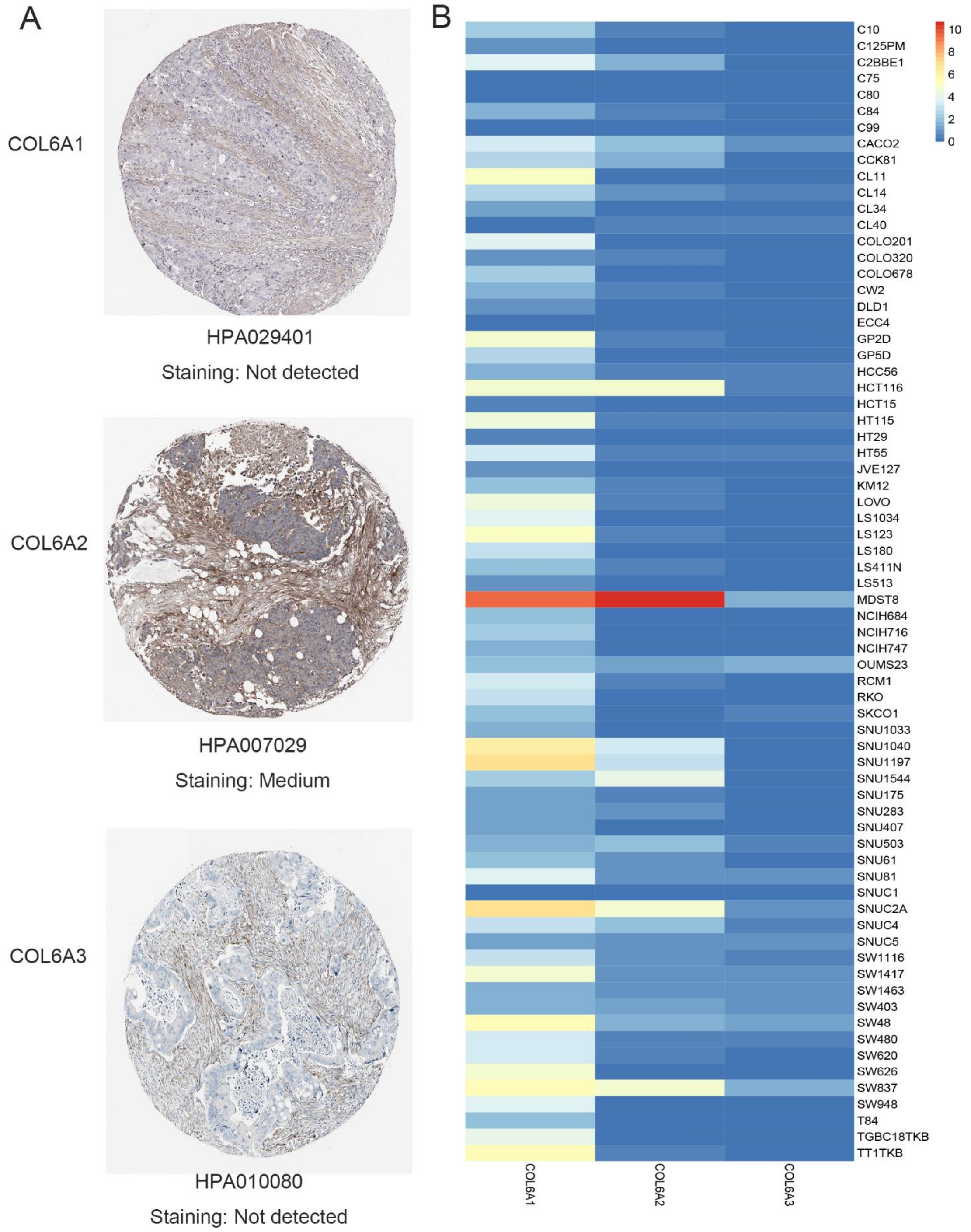

Supplement: Supplementary file 5 — Additional file 5. Expression of collagen VI family in colorectal cancer. [file 12859_2022_4951_MOESM5_ESM.pdf]
